# Electrothermal Denervation of Synovial and Capsular Tissue Does not Improve Postoperative Pain in Arthroscopic Debridement of Anterior Ankle Impingement—A Prospective Randomized Study

**DOI:** 10.1016/j.asmr.2021.11.019

**Published:** 2022-01-05

**Authors:** Sebastian Fischer, Sina Weber, Yves Gramlich, Marc Blank, Johannes Buckup, Sebastian Manegold, Reinhard Hoffmann

**Affiliations:** a Department of Foot and Ankle Surgery Berufsgenossenschaftliche Unfallklinik Frankfurt am Main, Frankfurt, Germany; b Department for Trauma and Orthopaedic Surgery Berufsgenossenschaftliche Unfallklinik Frankfurt am Main, Frankfurt, Germany; c Department of Sporttraumatology–Knee and Shoulder Surgery Berufsgenossenschaftliche Unfallklinik Frankfurt am Main, Frankfurt, Germany

## Abstract

**Purpose:**

The purpose of the study was to investigate the added value of electrothermal denervation (ETD) in arthroscopic debridement of anterior ankle impingement.

**Methods:**

Between May 2019 and December 2020, 58 patients who received arthroscopic anterior decompression for the impingement of the anterior tibiotalar joint were randomized to Group A (*n* = 29) with ETD of synovial and capsular tissue of the ankle and Group B (*n* = 29) without ETD. Patients included 37 men and 21 women, with a mean age of 42 years. The pain, range of motion (ROM), and function were recorded using the visual analog scale foot and ankle (VAS FA), the Foot Function Index (FFI), and the American Orthopaedic Foot and Ankle Society Score (AOFAS), both preoperatively and postoperatively.

**Results:**

Twenty-four hours after surgery, the pain level at rest using the VAS (worst 10 points) was 3.8 points on average (Group A: 3.7, Group B: 3.9). After 6 weeks, the mean VAS FA was 62.6 points, and ROM improved by an average of 9.1° (Group A: 9.8°, Group B: 8.6°; *P* > .05), the mean FFI was 40.4 points (Group A: 37.8, Group B: 42.8), the mean AOFAS was 73.1 points (Group A: 71.3, Group B: 75.1). All postoperative scores improved significantly compared with preoperative scores. No significant differences were observed between groups.

**Conclusions:**

The hypothesis of pain reduction with the use of ETD was refuted. The addition of ETD as part of the arthroscopic debridement of the anterior ankle impingement did not show any significant superiority in terms of the collected scores (VAS-FA, FFI, and AOFAS) at 24 hours and 6 weeks after the surgery and resulted in a comparable length of stay in the hospital and incapacity to work.

**Level of Evidence:**

Level I, prospective cohort study.

## Introduction

The genesis of anterior ankle impingement syndrome is typically traumatic, with the proliferation of bone and soft tissues, consisting of synovial membranes, fatty tissues, collagen, and vessels. In addition to various fractures of the ankle joint, ∼5% of all distortion traumas become chronic, with corresponding clinical symptoms.^1^, ^2^, ^3^, ^4^, ^5^ The present study refers exclusively to anterior ankle impingement. Typical anterior ankle impingement is caused by the repeated forced impact of the tibial crest against the neck of the talus or the soft tissue, resulting in the formation of scar tissue, which become impacted.^6^, ^7^, ^8^, ^9^, ^10^

Electrothermal denervation (ETD) describes the partial interruption of the sensitive nerve supply to the joint mucosa. A high-frequency current at the tip of the probe is guided by video-optical control to sclerotize the capsular and mucosal tissue along the front edge of the tibia and the neck of the talus. Although ETD of the synovium and the coagulation of small hemorrhages is an established procedure for the knee joint, especially peripatellar, the value of ETD at the anterior ankle has not yet been established.^11^, ^12^, ^13^

The purpose of the study was to investigate the added value of ETD in arthroscopic debridement of anterior ankle impingement. We hypothesized that reduced postoperative pain would enable the patient to mobilize more quickly and maintain the surgically improved ankle range of motion (ROM).

## Materials and Methods

### Ethical Review

All procedures were performed in accordance with the 1964 Helsinki Declaration and its later amendments or comparable ethics standards. The Ethics Committee of the Institutional Review Board approved this study (FF16/2019; DRKS00025703).

### Population

**Fig 1:**
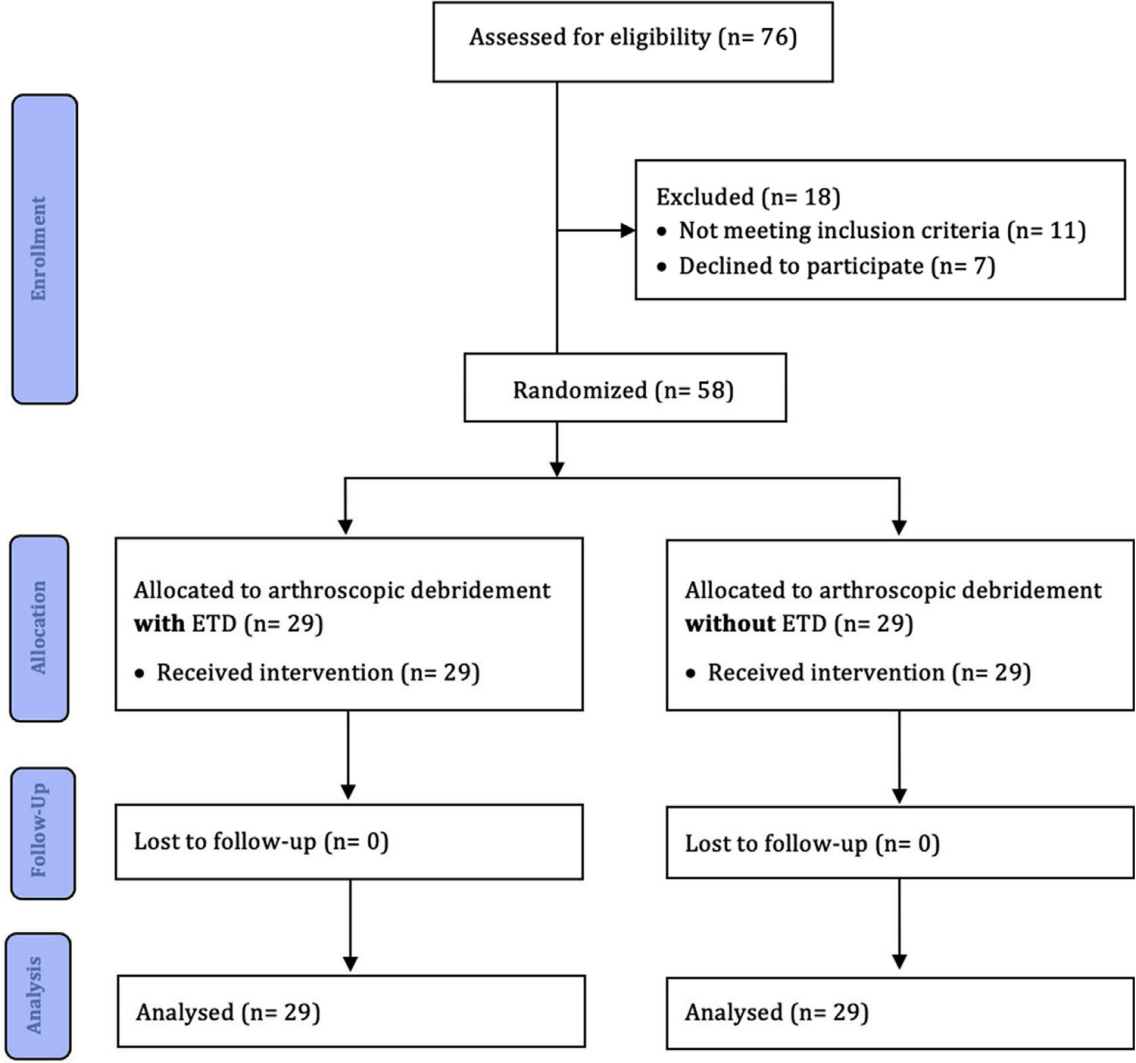
Study flowchart.

**Fig 2:**
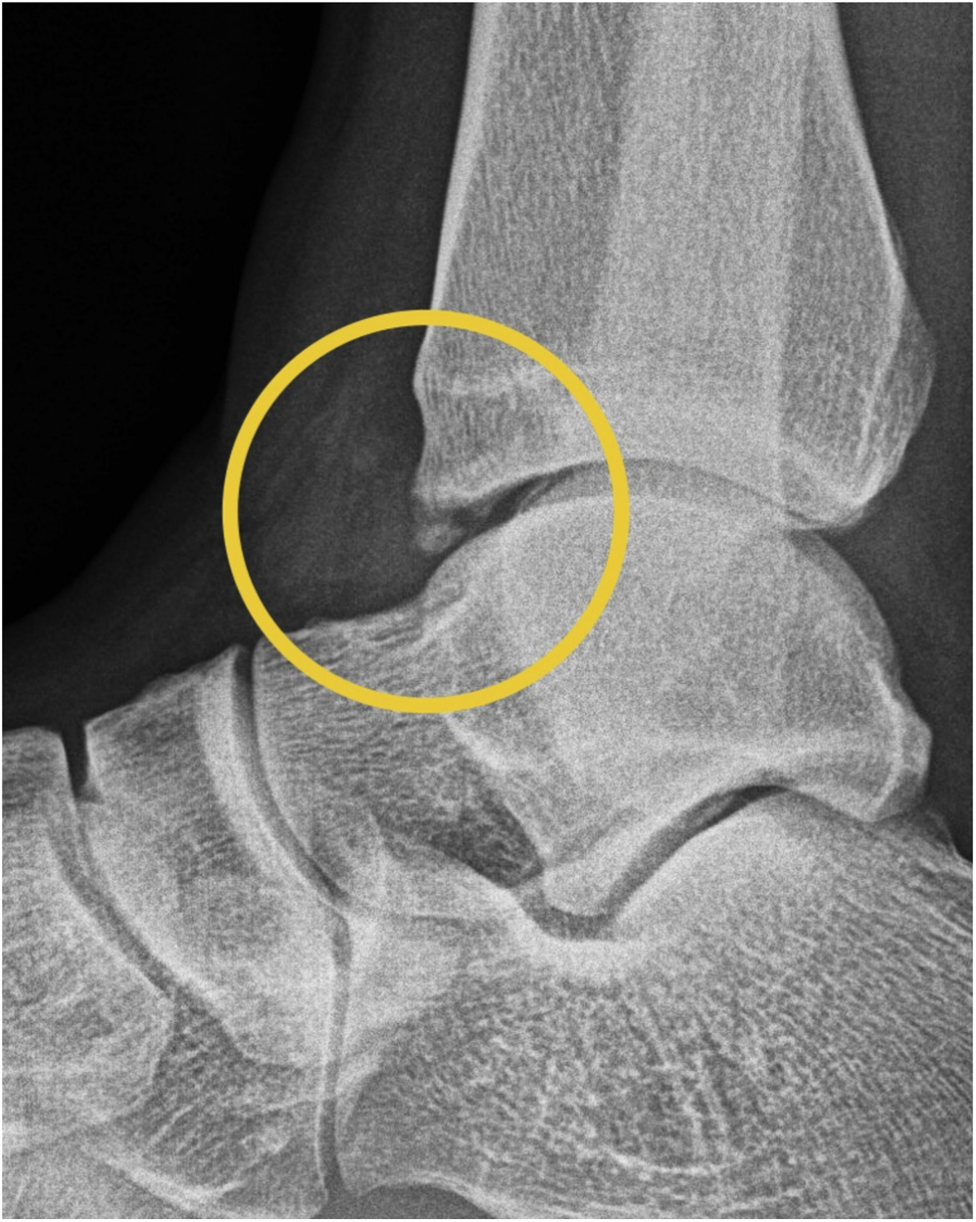
Imaging. Typical findings of radiographic imaging (Scranton and McDermott classification grade 2). Lateral view. Right ankle.

**Fig 3:**
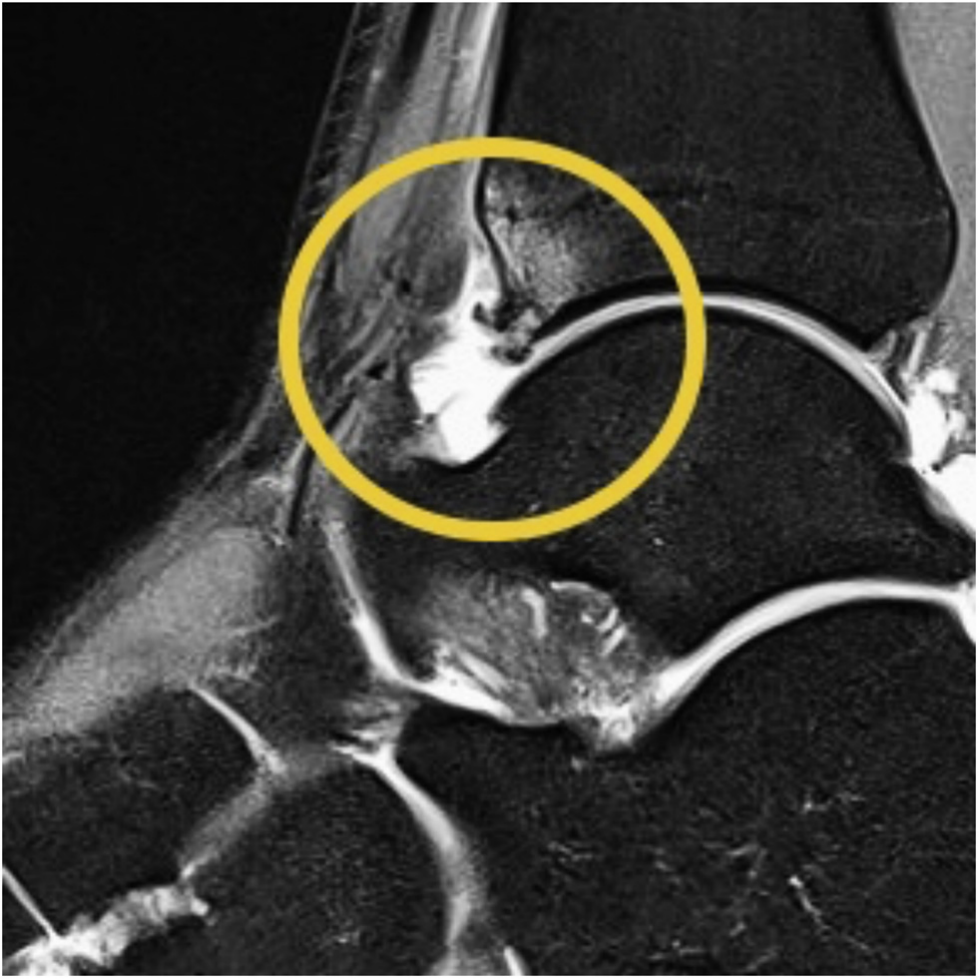
Imaging. Typical findings of magnetic resonance (MR) tomographic imaging (Scranton and McDermott classification grade 2). Sagittal view. Right ankle.

Between May 2019 and December 2020, 58 patients (37 men [63.8%], 21 women [36.2%]; mean age: 42 years [range: 19–70 years]) were included (Table 1, Fig 1). All patients included in this study had comparable demographic data (Table 1). Only the body mass index (BMI) differed. However, an influence on the results could not be determined. Patients were followed up for 6 weeks after all patients underwent arthroscopic debridement of anterior ankle impingement with ETD (Group A; *n* = 29) and without ETD (Group B; *n* = 29). All patients undergoing an arthroscopic debridement of the ankle joint were recruited during consultation at the study center. The diagnosis of anterior ankle impingement was made on the basis of a clinical examination and the results of radiographic and magnetic resonance (MR) tomographic imaging, depicted in Fig 2, Fig 3.

**Table 1:** Patient Characteristics (With Subgroups)

Characteristics	With ETD (*n* = 29)	Without ETD (*n* = 29)	All (*n* = 58)	*P*
Age (years)				
Mean (SE)	42.83 (2.46)	41.59 (2.37)	42.21 (1.70)	.718
Minimum	19.00	23.00	19.00	
Maximum	70.00	69.00	70.00	
BMI (kg/m^2^)				
Mean (SE)	28.71 (.93)	26.06 (.65)	27.39 (.59)	.023
Minimum	20.28	20.23	20.23	
Maximum	39.35	34.94	39.35	
Sex				
Male, *n* (%)	18 (62.1)	19 (65.5)	37 (63.8)	.789
Female, *n* (%)	11 (37.9)	10 (34.5)	21 (36.2)	
Affected side				
Left, *n* (%)	14 (48.3)	16 (55.2)	30 (51.7)	.607
Right, *n* (%)	15 (51.7)	13 (44.8)	28 (48.3)	
Smoker				
Yes, *n* (%)	8 (27.6)	7 (24.1)	15 (25.9)	.769
No, *n* (%)	21 (72.4)	22 (75.9)	43 (74.1)	
Pre-existing conditions				
Metabolic syndrome-associated, *n* (%)	3 (10.3)	7 (24.1)	10 (17.2)	.179
Others, *n* (%)	4 (13.8)	6 (20.7)	10 (17.2)	
None, *n* (%)	22 (75.9)	16 (55.2)	38 (65.6)	

BMI, body mass index; ETD, electrothermal denervation, SE, standard error.

The time between symptom onset and surgery ranged from 6 to 25 months, with the median time of 9 months. All procedures were performed in accordance with the 1964 Helsinki Declaration and its later amendments or comparable ethical standards. The ethics committee of the institutional review board approved this study, and the Consort guidelines were followed for this study. Informed consent was obtained from all patients.

### Inclusion and Exclusion Criteria

Patients with a minimum age of 18 years were included, with no maximum age. Written informed consent was required prior to participation in this study. All patients underwent pure arthroscopic debridement to treat the anterior ankle impingement.

Patients with generalized arthrofibrosis, higher-grade arthritic changes (2° up to 2 cm^2^, no grade 3 or 4 Outerbridge changes), and relevant ligament instabilities (>2°) of the tibiotalar joint were excluded. Patients with known coagulation disorders or at an increased risk of thromboembolism, patients undergoing permanent pain therapy, previous arthroscopic debridement of anterior ankle impingement, and patients with body mass index (BMI) >35 were also excluded.^14^

Any change of procedure to open arthrotomy and the perioperative or postoperative placement of a peripheral nerve catheter also resulted in exclusion. Randomization was performed using an urn model (29 per patient group). The surgeon was always allowed to perform or omit the ETD, regardless of the study-related group assignment. In the present study, this case did not occur.

### Surgical Procedure

**Fig 4:**
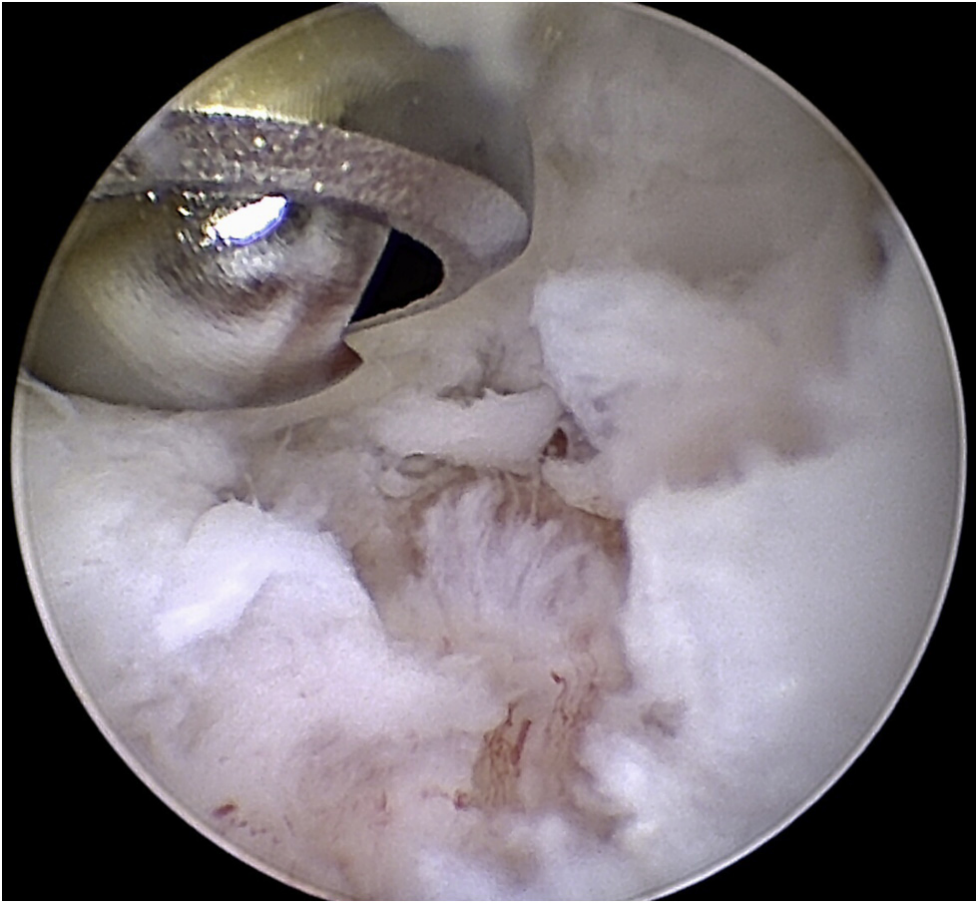
Intraoperative findings. Arthroscopic decompression of the anterior ankle impingement. Right ankle.

**Fig 5:**
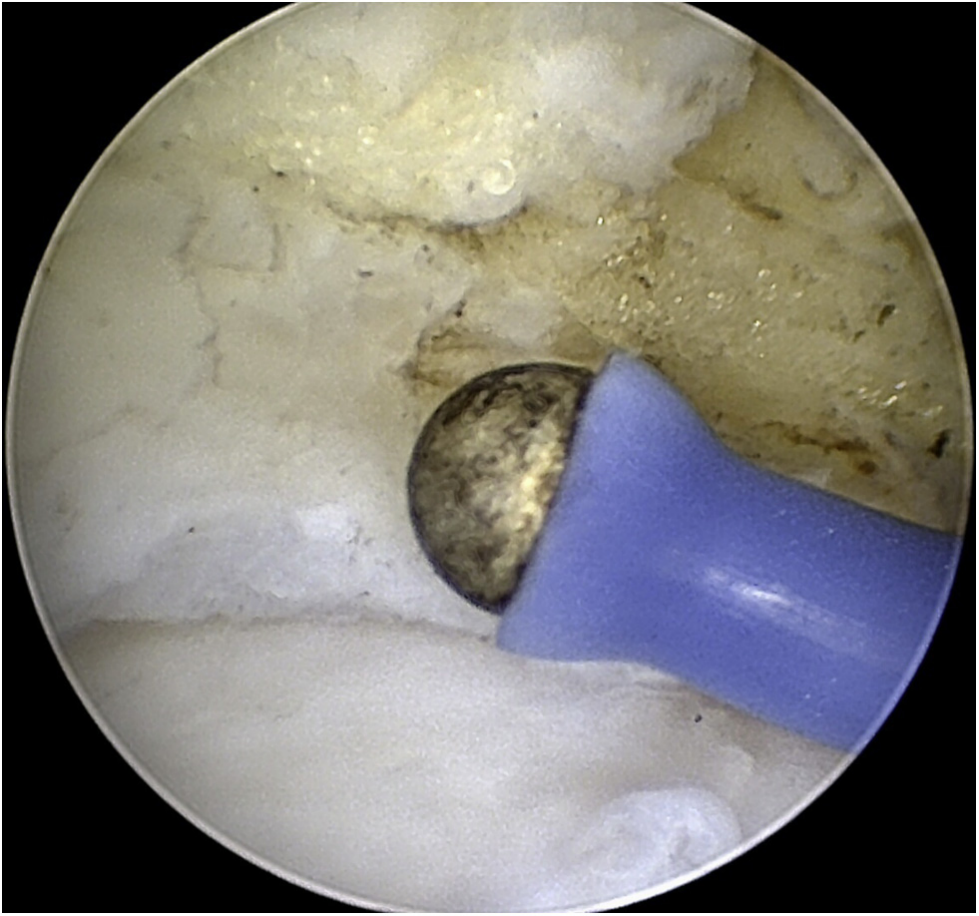
Intraoperative findings. Electrothermal denervation of synovialis. Right ankle.

Arthroscopy has established itself as the standard procedure for the anterior ankle and can be performed under regional or general anesthesia, using a shaver in an oscillating mode.^15^, ^16^, ^17^ For arthroscopic debridement of the anterior ankle, portals were created anteromedially and anterolaterally on the anterior ankle joint after closing the obligatory femoral tourniquet. After video-optical inspection, a shaver with a 3.0–3.8-mm diameter in oscillating mode was used for the resection, in addition to micro forceps (Fig 4). In patients with additional ETD, ETD was performed semicircularly along the anterior tibial edge and along the talus neck at the transition from the bone to the synovial membrane (OPES CoolCut Radio Frequency Small Joint Ablation device, Arthrex - in coagulation mode). Contact with the hyaline articular cartilage should be strictly avoided due to the potential for thermal damage (Fig 5).^18^^,^^19^ Irrigation fluid was standardized to a pressure of 40 mmHg. Before inserting the suction drain (drain size: 10 CH) over the medial portal, all irrigation fluid was aspirated. The drain was removed 24 hours postoperatively.

### Rehabilitation Protocol

The post-treatment protocol allowed for pain-adapted full weight bearing immediately after drain removal 24 hours postoperatively. All patients received the same standardized medication consisting of NSAIDs (nonsteroidal anti-inflammatory drugs). Steroids, local injections, or nerve catheters were not used.

For 5 days, patients used the aid of forearm crutches. Dorsiflexion and plantar flexion exercises were immediately initiated under physiotherapeutic guidance and were recommended to be continued daily for 6 weeks.

### Assessment Methods

As primary success parameters, preoperative and postoperative pain levels were evaluated using the visual analog scale (VAS). The preoperative and postoperative ROM was also compared using the neutral zero method. Demographic and health data, including BMI, preexisting conditions, such as diabetes mellitus and arterial hypertension (metabolic syndrome-associated), and nicotine abuse, were obtained for each patient. At the six-week postoperative follow-up, the VAS foot and ankle (VAS FA) scores, the Foot Function Index (FFI), in the German language-validated form (FFI-D), and the American Orthopaedic Foot and Ankle Society Score (AOFAS) were measured, and the type and number of complications were recorded. The output in the suction drain was also investigated.

The consultation and examination after 6 weeks were carried out by the treating surgeon during the consultation hours.

### Statistical Analysis

The study was designed as a monocentric, prospectively randomized, and single-blinded study. The randomization was performed by means of an urn model (29 per patient group). All procedures were performed by three experienced surgeons with equivalent expertise using the same technique. All statistical analyses were conducted using IBM SPSS Statistics version 23 (IBM, Ehningen, Germany). Mean values were compared using an independent-samples Student’s *t*-test. For the VAS, the minimal clinically important difference (MCID) was calculated to be two points on a scale that ranges from 0 to 10 points. The sample size calculation was based on the assumption of a two-point reduction in the VAS scale value 48 hours postoperatively when ETD was used during arthroscopic impingement debridement compared to without ETD. On the basis of an α-risk of .05, and a power of 80%, the number of cases required for this study was determined to be 58 (29 per group). The significance level of the results was set at *P* < .05.

## Results

Fifty-eight patients were followed up; there were no cases lost to follow-up. On the first day after surgery, the pain level at rest using the VAS (worst 10 points) was 3.8 points on average (Group A: 3.7, Group B: 3.9). The difference between groups was not significant (*P* = .230) (Table 2).

**Table 2:** Outcome 24 and 48 Hours Postoperative (With Subgroups)

Measurements	With ETD (n = 29)	Without ETD (n = 29)	All (n = 58)	p
VAS of pain at rest 24 hrs post-op				
Mean (SE)	3.72 (.46)	3.91 (.48)	3.81 (.33)	.782
Minimum	.40	.70	.40	
Maximum	9.30	9.10	9.30	
VAS of pain at rest 48 hrs post-op				
Mean (SEM)	2.57 (.35)	2.05 (.40)	2.31 (.26)	.332
Minimum	.20	.00	.00	
Maximum	7.00	7.50	7.50	
VAS of pain in activity 48 hrs post-op				
Mean (SEM)	3.61 (.34)	3.75 (.46)	3.68 (.29)	.808
Minimum	.20	.60	.20	
Maximum	7.30	9.80	9.80	
Operating time (min)				
Mean (SEM)	51.14 (3.95)	45.52 (3.05)	48.33 (2.50)	.265
Minimum	23.00	13.00	13.00	
Maximum	119.00	80.00	119.00	

ETD, electrothermal denervation; hrs, hours; min, minutes; post-op, post-operative; SE, standard error; VAS, visual analog scale (worst 10 points).

**Fig 6:**
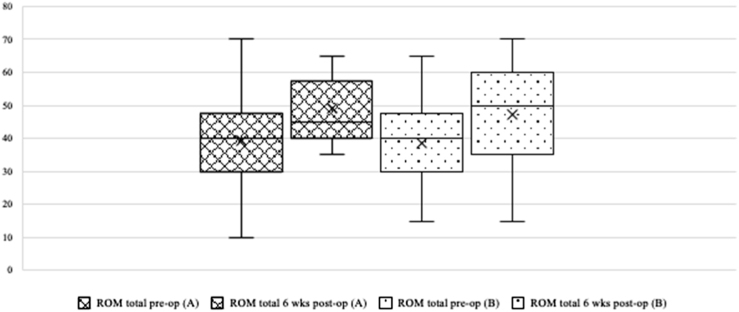
Outcome range of motion, preoperative and 6 weeks postoperative. Group A with ETD; Group B without ETD; ETD, electrothermal denervation; ROM, range of motion (in °); pre-op, pre-operative; post-op, post-operative; wks, weeks.

**Fig 7:**
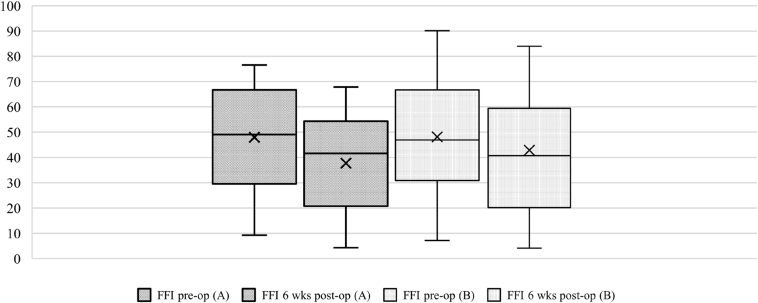
Outcome Score FFI, preoperative and 6 weeks postoperative. Group A with ETD; Group B without ETD. ETD, electrothermal denervation; FFI, Foot Function Index (worst 100 points); pre-op, pre-operative; post-op, post-operative; wks, weeks.

After 6 weeks, the mean VAS FA was 62.6 points (Group A: 62.61, Group B: 62.6; *P* = .998), the mean AOFAS value was 73.1 points (Group A: 71.3, Group B: 75.1; *P* = .585), and the mean FFI score was 40.4 points (Group A: 37.7, Group B: 42.8; *P* = .523) (Table 3). The total ROM for the ankle joint improved on average from 39.1° to 48.1° (*P* = .684). Dorsiflexion and plantar flexion benefited equally (Fig 6). All scores and the ROM improved significantly between the preoperative and postoperative assessments, including highly significant changes in AOFAS ankle-hindfoot (VAS FA, *P* = .005; FFI, *P* = .046; AOFAS-AH, *P* = .058), and ROM (*P* = .027) (Fig 7). Group allocation had no significant influence on any measurement (Table 3). The average operation time was 5.6 minutes longer with the use of ETD (Group A: 51.1 minutes, Group B: 45.5 minutes), but the difference was not significant (*P* = .265). The mean output in the suction drain after 24 hours was 59.6 mL (Group A: 49.4 mL, Group B: 69.4 mL); the difference was not significant (*P* = .233).

**Table 3:** Outcome Preoperative and Six Weeks Postoperative (With Subgroups)

Measurements	With ETD (*n* = 29)	Without ETD (*n* = 29)	All (*n* = 58)	*P*
VAS FA (max. 100 points) pre-op				
Mean (SE)	50.27 (3.75)	53.10 (3.56)	51.68 (2.57)	.586
Minimum	19.60	19.40	19.40	
Maximum	84.11	90.05	90.05	
VAS FA (max. 100 points) 6 wks post-op				
Mean (SE)	62.61 (5.01)	62.62 (5.39)	62.614 (3.66)	.998
Minimum	27.05	30.16	27.05	
Maximum	89.95	95.30	95.30	
AOFAS-AH (max. 100 points) pre-op				
Mean (SE)	66.31 (1.93)	68.14 (2.69)	67.22 (1.64)	.583
Minimum	42.00	31.00	31.00	
Maximum	85.00	88.00	88.00	
AOFAS-AH (max. 100 points) 6 wks post-op				
Mean (SE)	71.25 (4.87)	75.13 (5.08)	73.13 (3.47)	.585
Minimum	34.00	37.00	34.00	
Maximum	100.00	98.00	100.00	
FFI (max. 100 points) pre-op				
Mean (SE)	47.94 (3.87)	48.14 (4.11)	48.04 (2.80)	.972
Minimum	9.26	7.19	7.19	
Maximum	76.54	90.12	90.12	
FFI (max. 100 points) 6 wks post-op				
Mean (SE)	37.72 (5.04)	42.82 (6.00)	40.35 (3.91)	.523
Minimum	4.32	4.17	4.17	
Maximum	67.90	83.95	83.95	
ROM (°), mean pre-op				
Total (SE)	39.48 (2.51)	38.62 (2.39)	39.05 (1.72)	.805
Dorsiflexion	9.83	8.28	9.05	.313
Plantar flexion	31.03	30.35	30.70	.787
ROM (°), mean 6 wks post-op				
Total (SE)	47.69 (1.83)	45.44 (3.82)	46.63 (2.02)	.583
Dorsiflexion	12.38	12.78	12.50	.823
Plantar flexion	35.36	34.77	35.10	.801

AOFAS-AH, American Orthopaedic Foot and Ankle Society Ankle-Hindfoot Scoring System (best 100 points); ETD, electrothermal denervation; FFI, Foot Function Index (worst 100 points); pre-op, pre-operative; post-op, post-operative; ROM, range of motion; SE, standard error of the mean; VAS FA, visual analog scale foot and ankle; wks, weeks.

The average length of hospital stay was 2.2 days (Group A: 2.2 days, SE: .16; Group B: 2.1 days, SE: 0.21). A tendency of slightly prolonged incapacity to work in the group with ETD was observed (Group A: 33 days, SE: 2.5; Group B: 29 days, SE: 3.4). The difference between groups was not significant (*P* > .05).

### Complications

In group A, one patient complained of paresthesia along the dorsum of the foot in the area supplied by the superficial peroneal nerve. In group B, one patient complained of neuropathic pain in the anterior ankle joint. Both symptoms subsided after 6 weeks. No complications of skin or wound healing from thermal ablation were observed.

## Discussion

The most important finding of the present study was that with regard to the main objective criteria of pain reduction within the first 48 hours after surgery, the use of ETD did not show any positive effects. The VAS FA, FFI, and AOFAS scores, which were re-evaluated after 6 weeks, also showed comparable results between the two groups, tabulated in Table 2, Table 3 and Fig 7.

Numerous studies have explored the topic of anterior ankle impingement, especially the development of this phenomenon. The primary factors, proliferation of bone and soft tissues, consisting of synovial membranes, fatty tissues, and collagen, have been identified.^9^^,^^20^^,^^21^ The outcome after debridement is also a frequent subject of current literature,^22^ whereby arthroscopic debridement is preferred over open debridement.^10^^,^^23^^,^^24^ Remarkably, the benefits of ETD have not been discussed to date.

Orhurhu et al. attributed positive effects to radiofrequency treatment of the ankle for the management of chronic foot and ankle pain. It should be noted that the collected studies were not designed prospectively.^25^ The review’s summary could not be confirmed by the present study.

Although the additional ETD, which was established as a circumferential denervation of the patella during primary total knee arthroplasty, is an established procedure during knee operations, for example, no clear recommendations have been established for the use of ETD in the ankle joint.^11^^,^^12^ The results of the present study refute this assumption, that vessel sclerotherapy during the course of arthroscopy for the ankle joint would also result in a simultaneous reduction in pain.

Hemarthrosis is an important cause of ankle pain. Therefore, regardless of the primary intention of the study, the output in the suction drain was also investigated. With regard to bleeding into the ankle joint, no significant difference could be documented on the basis of the measured flow rate 24 hours postoperatively. Similarly, no clear cause could be identified among the available data for those cases in which more postoperative bleeding into the tibiotalar joint was observed. All patients had normal laboratory parameters before surgery. A considerable proportion of possible bleeding may have been caused by portal placement. The extent to which the implementation of routine ultrasound monitoring can be implemented into everyday clinical practice, as recommended by Scheibling et al. to define landmarks during surgical preparation, should be explored.^26^

The results of the present study confirm arthroscopic debridement as an appropriate treatment for anterior ankle impingement. Studies with similar objectives show comparable results.^10^^,^^16^^,^^23^ All collected scores (VAS FA, FFI, and AOFAS) and the ROM improved significantly from preoperative to postoperative, which is highlighted in Fig 6, Fig 7.

However, group allocation had no effects on the outcomes. The following assumptions are made: The significantly positive effect of the intervention resulted in the reduction of disruptive scar tissue and bony attachments but not in the obliteration of the mucosa and the capsular tissue.^27^ No thermal damage to the skin or wound healing disorders could be detected as a result of ETD.

The overall low complication rate observed in the present study does not indicate a negative effect of the ETD, based on the early postoperative results. However, the uncritical use of ETD carries the potential risk of cartilage damage to the tibia and talus.^28^^,^^29^ Thus, routine use should be discouraged.

The risk of complications with ankle arthroscopy is generally low. However, complication rates of up to 9% are still too high and, in the authors’ view, can be avoided by using a soft-tissue-conscious technique, regardless of the procedure.^30^

In addition, the routine use of ETD in the absence of any objective benefit results in a tendency toward prolonged operation times. To be distinguished from this is the use of electrothermal shrinkage as a stand-alone treatment or as an adjunct to arthroscopic surgery. The present study did not examine the effectiveness of this intervention. Patients with relevant ligament instabilities of the tibiotalar joint were excluded.

The strengths of this study include in the number of cases—prospectively randomized, monocentric design, and homogeneity of the patient population.

### Limitation

However, some limitations of this study need to be considered. Despite its international use and good comprehensibility, the AOFAS has not yet been validated for the German language.

All patients were encouraged to continue intensified physiotherapy; however, self-exercise could not be observed or evaluated. In terms of the assessment methods—postoperative pain levels, postoperative range of motion, and the length of inpatient stay—a short follow-up period was chosen.

### Conclusion

The hypothesis of postoperative pain reduction with the use of ETD was refuted. The addition of ETD as part of the arthroscopic debridement of the anterior ankle impingement did not show any significant superiority in terms of the collected scores (VAS-FA, FFI, AOFAS) at 24 hours and 6 weeks, and resulted in comparable length of hospital stay and incapacity to work.

## Supplementary Data

ICMJE author discloser forms
